# Supplementary material for: Influence of trip distance and population density on intra-city mobility patterns in Tokyo during COVID-19 pandemic
Source: PLoS One. 2022 Oct 28;17(10):e0276741. doi: 10.1371/journal.pone.0276741 (PMC9616219; doi:10.1371/journal.pone.0276741)
Supplement: Attachment — Submitted filename: Response to Reviewers.pdf [file pone.0276741.s002.pdf]

Dear Dr. Itzhak Benenson

,

Thank you for sending the helpful comments from the reviewers. Considering them carefully, we fundamentally revised the paper. Following the comments from Reviewer 1, we also modified and re-calculated all estimation results and some new results. Additionally, we revised the introduction using new references so the paper's contribution is more apparent. The revised parts of the manuscript are indicated by blue text.

Detailed replies to each reviewer are as follows. We appreciate your consideration of them with the revised paper.

Reply to the comments from Reviewer #1:

Thank you so much for your really helpful suggestions on reliability of the model's prediction for small variables. We modify all the estimation following them, although the results did not change significantly. We also add more detailed estimation and discussion for time series trend of the mobility Following your advices. The replies to each of them are as follows:

Q1.
The main issue starts from the specification eq 1. If one sets infections (IFC) to zero, then one obtains infinitely many trips. In consequence, this is not a specification one could just copy to be used in other models.

A1.
Although we actually used ln(1+IFC) in the submitted paper, it was not clearly mentioned. Therefore, the original model can be applied to the case with IFC=0. However, we conducted more elaborate model specification, following the manner mentioned in Q3;please see A3 for more details. Please refer to "Methodology" and "Results" in the manuscript.

Q2.
I think what has happened is that the authors have selected a model specification that is particularly suited for the determination of elasticities. This becomes clear in the (relatively short) section on "Total effects and their sensitivity". Elasticities are useful short-term constructs, i.e. they can answer questions such as:
Infections today are at level X, now let us assume that they grow by 50%, how will mobility change. But elasticities are not useful to describe everything from before the pandemics until after the second lockdown in one equation. To make the model useful, the authors need to move more into that direction, i.e. how elasticities change over the course of the pandemics. A first step is done in table 2. And here one sees how unstable the results are: While table 1 finds a 22% reduction in trips by the first lockdown, table 2 (which investigates the period up to and including the first lockdown separately), that reduction is now down to 16%. At the same time, the effect of each 1% more infections increases from 0.011% to 0.027%. Personally, I would try elasticities month by month,

but in the end that is up to the authors.

A2.

We agree with your comment. In reality, elasticity of mobility regarding new infection cases, (dP/dIFC)/(P/IFC), will differ depending on time and situation because of the following two reasons:

1. Sensitivity or coefficient ¥beta might change, although our basic analysis assumes that it is fixed throughout the data period.
2. Elasticity of X (e.g. number of new infection cases) depends on the current value of X if we employ the function ¥beta ln(1+X), although this problem does not matter when we use ln (X).

Since the reviewer mentions the first problem, we specifically focused on it. We tried separating the data periods into five segments, where the length of one segment is about three months. However, further detailed segmentation is hard to implement because our data point is only once a week (i.e., Thursday at 10:00 AM) to control the effect of day and time. Please see "Trend of trip patterns" in "Results." Figure 9 simply shows the "total effect" in each period, which is almost the same as the elasticity if X is sufficiently high (e.g., more than 100). Please see the "Trend of trip patterns."

For the second problem, we needed to calculate the elasticity for different X. However, we actually employed the function as ¥beta ln(1+X/0.5) for the number of infection cases, where 0.5 is set to maximize the within R2 of the model following Comment 3. Since X is much more than 100 in many cases, "1" in ln(1+X/0.5) is almost negligible. Therefore, the difference of X does not matter so much for the value of elasticity. Please see L290-291 and Footnote 15.

Q3.

A second issue is that the model specification is unstable even with respect to its signs. Model 1 from Table 1 makes sense, except for LD2 (= second lockdown), which, however, could as well just be set to zero. However, in Model 2 of the same Table, the coefficients of ln(ifc), ln(long), ln(ld1), ln(ld2) are now positive, implying (for example) that higher infection numbers mean more trips. This is, presumably, just there to compensate for some effect of the crossterms, but for a model that is useful in practice one should not use the lower order terms to correct for oddities in the higher order terms. (In this case, possibly the model is misspecified for small values of "distance" or "density".)

This goes along with the fact that distance and density (and some other things) need units. It then becomes clear that, for models that use logarithms, the units are also free parameters, i.e. presumably the model should be beta_dens * ln(dens/dens_*), where dens_* should also be estimated. Or maybe even beta_dens * ln(1 + dens/dens_*) to get rid of the singularity near zero. I am not a statistician, but, as stated, a model with as many oddities as the present model will be impossible to use in practice.

A3. To solve the problem in the model with the cross terms, we used the functional form ln(1 + x/¥omega^x), where x is IFC, Dens, or Dist. The unit of each variable, denoted by ¥omega^x, is chosen to maximize the within R^2 of the model. Because of the computational burden from our large dataset, we examined 27 patterns (3*3*3 patterns) of ¥omega^x and chose the best one among them). Please see "Methods," especially L232-238.

However, even after considering such flexible specification, the problem is not completely solved; the effect of IFC is positive for about 17% OD samples in our dataset with short distance and low density (i.e., about 5km and 1000pop/cell). Although this seems counterintuitive, some previous research also indicated that people have shifted to low-density and short-distance trips, so we mentioned them to support our result. Please see L312-315.

Further, for LD 1&2, no positive significant effect is predicted for any OD samples, unlike IFC. This result may be explained by the "intervention effect" of compulsory lockdown as well as the information effect, as mentioned in Watanabe and Yabu (2021). Please see L319-321.

Minor:

Q4.

Why are IFC and LONG not divided by the population size? By not dividing them, one pretends that having 300 infection cases in a population of 3000 is the same as having it in a population of 30'000.

A4. Following your suggestion, we defined IFC by using the number of new infection cases divided by relative population in order to control the difference of population size among prefecture. The relative population size of prefecture *i* is defined by (population of prefecture i)/(population of Tokyo), and the number of infections is divided by the relative population. The absolute value of population is absorbed when we choose the

unit of population. However, such control of population is not necessary for LONG because it is the ration of infection cases between last week and two weeks ago; hence, population difference does not matter. Please see L214-215.

Q5.
Fig 5a and 5b look the same to me.
A5.
This is our mistake, so we replaced the figures with the correct ones.

Q6
I would like to see the resulting alpha_t (or exp(alpha_t)) after the estimations where applicable (e.g. Table 1 models 3 and 4). One would expect that this will trace overall mobility quite well.

A6
We check the estimates of the data fixed effects, and show them in Figure 8. The trend of fixed effect shows how the basic mobility of people changes by period. Although the effect of the two lockdowns are also included in them because they are not separable(so we omit LD1&2 from Model 3,4 in table 1) as mentioned in the mainbody of the paper, they also reveals some hidden attitudes and trend regarding mobility.

The findings from the date fixed effects are as follows. First, alpha_t gets exceptionally low during the first lockdown because of the effect of LD1 in it.
Second, alpha_t is continuously high in the latter half of the data. In these periods, it is considered that people are used to the pandemic.

Reply to the comments from Reviewer #2:

Thank you for your suggestion regarding related literature that makes our contribution clearer. They were helpful for revising the Introduction. Other detailed comments on our writing were also helpful for clarification, so we considered each comment carefully. The replies to each of them are as follows:

1) L4, L11: "according to" not needed in both cases

A. We omitted this phrase per your suggestion.

2) L16-L19: The two sentences here need to be better connected. There needs to be a better transition explaining why economic reasons (and not epidemiological reasons) necessitate the study of changes in daily mobility by lockdowns.

A. We added sentences to mention why we focused on compulsory lockdowns and people's "behavior" from the viewpoint of economic damage. Please see the second paragraph of the Introduction. (L17-21)

3) L25: What is meant by "Place IQ"? do you mean PlaceIQ.com?

A. We omitted the original sentence which included the term when we revised the second paragraph of the Introduction.

4)L38-L45: Actually, there have been quite a few studies using cellphone data to study changes in daily mobility during the COVID-19 pandemic, including ones that looked at spatial patterns of mobility. I suggest you do a more extensive literature search and identify more accurately the research gaps that you are responding to, and hence the novelty of the paper.

A. We added and referred to several previous studies that use cellphone data. Although mobile phone data has been used by a growing number of recent studies, not as many studies focus on decision-making regarding trips during the COVID-19 pandemic; hence, our contribution is to add new results to the literature. Please see the third paragraph of the Introduction around L18-33.

5) L52-L53: The phrase "they capture people's hourly location at 500m x
500 m cell level with their residency" is not clear. Do you mean "within
their residency"? I suppose you meant to say that the cellphone data
have been aggregated to grid cells of 500 m resolution, but what does

the residency do here?

A. We changed the sentence as: “Our data, collected and estimated from cellphones, are suitable for investigating intra-city trips because they capture how many people who have residency in each city are within a 500 x500 m cell each hour.”(L58-59) Since the definition of the data may be difficult to understand for most readers, we added a new figure (Fig. 2) to show the structure of the dataset.

6) L64: “Summary of” not needed.

A. We omitted these words.

7) Figure 1: The line width used is too heavy, which particularly renders 1B illegible (the curve for Saitama is not visible).

A. We made the lines thinner, though identifying the differences among the prefectures in the early stage of the pandemic is still difficult due to the scale of the figure.

8) L74: "Review of" not needed.

A. We omitted the words.

9) L114: After "was the same", insert a reference to Fig. 1B.

A. We revised the sentence as “*…the period of quasi-lockdown was the same as shown in Fig. 1B.” (L121)*

10) L135: Is NHK the Japanese government health agency? Needs some explanation.

A. NHK is an acronym for Nippon Hoso Kyokai” (Japan Broadcasting Corporation), which is the public broadcaster in Japan. Please see L143

11) L143: "the QGIS" --> "QGIS"

A. We revised this according to your suggestion.

12) L152: "time zone" is ambiguous.

A. We changed the sentence to “*Although the objectives of trips are not available in our data, we are especially interested in commuting trips, and measuring the temporary population at 10:00 AM is appropriate because most people finish commuting by then.*”(L160-161)

13) L163: "reasonability" sounds awkward in this context.

A. We revised as follows: “To check the validity of this idea, we also show the locations at 9:00 and 11:00.”(L171)

14) Figure 4: This figure has several cartographic problems:

- The figure-ground relationship is not clear. What is sea, what is land? For instance, using a shaded relief map in the background could clarify that.
- The figure needs a scalebar.
- The city limits of Tokyo should be shown. Same for the study area, if the study area extends over the city limits.
- Additional labelling, or adding a shaded relief map in the background, could provide better spatial reference. For non-Japanese readers, it's hard to understand what we see here.

A. We revised Figure 5(number of the figure changes) to show the geography of Japan and the targeted area clearly.

15) L182: Why 4000 people? Why this number?

A. The number (4000 people) is obtained from looking at Figure4, and we never use 4000 for a threshold in the later analysis except for Figure5. So we revised the sentence as: “Fig 5 shows that change in mobility occurs in grid cells with high population density, which seems to be especially significant for a populations larger than 4000.”(L188-190)

16) Figure 5: Fig. 5A was included twice, while Fig. 5B was missing in the manuscript. However, the correct figure was contained in the file Figs.pdf that was available for download.

A. This is our mistake, and we corrected Figures 6 a,b. (number of the figure changes).

17) Figure 5 is too small to understand what's happening here. I suggest adding an inset map showing a zoomed-in portion that is particularly interesting for the story of the paper.

A. We presented a zoomed in map to show what happens around the central area of Tokyo MA. Figure 6 clearly shows that lockdown significantly affects the center rather than the

subcenter; hence, the influence of distance and density might be anticipated in a very indirect manner, though we need to show clearer and more direct evidence. Please also see L200-205, which mentions how this figure relates to our story.

18) L196: Change this title to "Methods"
A. We changed in this way. The new table of contents is as follows (the new sections are colored in blue):

- Introduction
- Background
  - COVID-19 in Tokyo
  - Lockdowns in Japan and other countries
- Data
  - Data
    - Mobile spatial statistics data
    - The number of COVID-19 cases
    - Geographical data
    - The data period and targeted area
  - Summary of the mobility data
    - Patterns of morning trips
    - The effect of quasi lockdowns
- Methods
- Results
  - Baseline estimation
  - Total effects and their sensitivity
  - Trend of trip patterns
  - Mobility in weekend afternoon
- Conclusion

19) L213: What does the "it" in "Third, it also includes" refer to?
A. "it" means variable X_{ij,t}. Hence, we clearly mention that in this sentence. (L225)

20) L220:
- Before L220, insert a new section title "Results". (Same heading
hierarchy level as "Methods")
- Change "Results of the baseline estimation" to "Baseline estimation"

A. We revised the titles of the sections in this way. Please see the answer to Comment 18 above.

21) L229: I can't see 22.2% and 0.26% in Table 1.
A. We changed these numbers as we changed the estimation results. The revised numbers are 21.92 and 0.28 as in Table 1. Please see L250.

22) Footnote 12: Sections are not numbered. Hence, the reference to Section 3.1 won't work.
A. Instead of "Section 3.1," we stated, "in the section titled Total effects and sensitivity." Please see Footnote 13.

23) Footnote 13: change "unlike" to "except"
A. Since the original sentence was unclear, we changed it to: "Note that LDs are dummies and hence take the values 1 or 0, while IFC is a continuous variable whose natural logarithm is used for the independent variable." Please see Footnote 16.

24) L276: change "almost negative" to "slightly negative"? "almost" seems odd.
A. We revised this sentence as: "*They show that the total effect of IFC is negative in most cases in Figure 5, except for a case with small population density.*"(L299)

25) Footnote 17: September 2020
A. This footnote was omitted in the revised paper.
