# Supplementary material for: Influence of trip distance and population density on intra-city mobility patterns in Tokyo during COVID-19 pandemic
Source: PLoS One. 2022 Oct 28;17(10):e0276741. doi: 10.1371/journal.pone.0276741 (PMC9616219; doi:10.1371/journal.pone.0276741)
Supplement: Attachment — Submitted filename: [10.7 ver2] Response to Reviewers.pdf [file pone.0276741.s003.pdf]

Dear Dr. Itzhak Benenson,

Thank you for sending the helpful comments. Considering them carefully, we revised the paper. The revised parts of the manuscript are indicated by blue text in the revised manuscript with track changes.

Reply to the comments from Academic Editor

Q. *Please review your reference list to ensure that it is complete and correct. If you have cited papers that have been retracted, please include the rationale for doing so in the manuscript text, or remove these references and replace them with relevant current references. Any changes to the reference list should be mentioned in the rebuttal letter that accompanies your revised manuscript. If you need to cite a retracted article, indicate the article's retracted status in the References list and also include a citation and full reference for the retraction notice.*

A. References were revised following Vancouver style as indicated in the Submission Guidelines: https://journals.plos.org/plosone/s/submission-guidelines. We confirmed that no papers were retracted.

Reply to the comments from Reviewer

Q1, p6, line 183: An overleaf URL has sneaked in, presumably by erroneous copy/paste.

A. Thank you so much for your comments. The following URL was removed from the revised manuscript: https://ja.overleaf.com/project/6138170906460e123b1296d5. We made sure there were no mistakes elsewhere.

Q2, The Fig.10 that I have in the submission is not consistent with the description: I think that this duplicates Fig.8(B) instead of showing something about the separate periods.

A. The Fig.10 was replaced with the correct one.

In addition to the reviewer’s comments, we made the following additional revisions to the manuscript.

・p.8 line 229-230: The text was revised to be more accurate.

[**before**] "Single terms such as distance, density, and 229 lockdown dummies in few results are excluded because they are included in the fixed effects of OD and date."

[**after**] "Single terms of $Dens$ and $Dist$ are excluded because they are included in the fixed effects of OD. In the same reason, $LD1$ and $LD2$ are excluded when the date fixed

effects are estimated."

Several other minor corrections were made.

・Periods (.) and hyphens (-) was added in several places.

・"IFC", "LONG", and "LD" fonts were unified.

・p.1 line 3: The number of infected persons was corrected.
[before] 400 million -> [after] 200 million

・p.7 line 227: There were two periods, so we removed one of them.
